# A cross-sectional analysis of the associations between leisure-time sedentary behaviors and clustered cardiometabolic risk

**DOI:** 10.1186/s12889-018-5213-3

**Published:** 2018-03-06

**Authors:** Antje Ullrich, Lisa Voigt, Sophie Baumann, Franziska Weymar, Ulrich John, Marcus Dörr, Sabina Ulbricht

**Affiliations:** 1 University Medicine Greifswald, Institute of Social Medicine and Prevention, Walther-Rathenau-Str. 48, D-17475 Greifswald, Germany; 2 German Centre for Cardiovascular Research (DZHK), partner site Greifswald, Fleischmannstr, 42-44, D-17475 Greifswald, Germany; 3 University Medicine Greifswald, Institute for Community Medicine, Section Epidemiology of Health Care and Community Health, Ellernholzstr, 1-2, D-17487 Greifswald, Germany; 4 University Medicine Greifswald, Department of Internal Medicine B, Ferdinand-Sauerbruch-Str, D-17475 Greifswald, Germany

**Keywords:** Sedentary behavior, Cardiometabolic risk, Linear regression, Quantile regression, Metabolic syndrome, Multiple imputation

## Abstract

**Background:**

The aim of this study was to conduct a comprehensive investigation of the association between different types of leisure-time sedentary behavior (watching television, using a computer, reading and socializing) and clustered cardiometabolic risk in apparently healthy adults aged 40 to 65 years.

**Methods:**

One hundred seventy-three participants from the general population (64% women; mean age = 54.4 years) consented to attend a cardiovascular examination program and to complete a questionnaire on leisure-time sedentary behaviors. Waist circumference, blood pressure, glucose, triglycerides, and high-density lipoprotein cholesterol of non-fasting blood samples were assessed, and a clustered cardiometabolic risk score [CMRS] was calculated. Data were collected between February and July 2015. Associations between leisure-time sedentary behaviors and CMRS were analyzed using linear and quantile regression, adjusted for socio-demographic variables and other types of leisure-time sedentary behavior (model 1) and additionally, adjusted for leisure-time physical activity and traveling in motor vehicles (model 2).

**Results:**

Linear regression revealed that there was a positive association between watching television and CMRS (model 1: b = 0.27 [CI: 0.03; 0.52]; model 2: b = 0.30 [CI: 0.05; 0.56]). In addition, quantile regression analysis revealed that using a computer was negatively associated with the 50th (model 1: b = − 0.43 [CI: -0.79; − 0.07]) and the 75th percentiles (model 1: b = − 0.71 [CI: -1.27; − 0.14]) of CMRS. Reading and socializing were not associated with CMRS.

**Conclusions:**

Watching television was positively associated with a clustered cardiometabolic risk score, while time spent using a computer revealed inconsistent findings. Our results give reason to consider different types of behaviors in which individuals are sedentary and the associations between these behaviors and cardiometabolic risk, supporting the need for behavior-specific assessments as well as public health recommendations to maintain or enhance adults’ health.

**Trial registration:**

Clinical trial registration number: NCT02990039, Retrospectively registered (December 12, 2016).

**Electronic supplementary material:**

The online version of this article (10.1186/s12889-018-5213-3) contains supplementary material, which is available to authorized users.

## Background

Sedentary behavior [SB], defined as any waking behavior characterized by an energy expenditure ≤ 1.5 metabolic equivalents while in a sitting or reclining posture [1], is highly prevalent [2]. Prevalence has increased as utilization of media technologies in leisure time has increased. Physical activity [PA] has decreased, both at work and in leisure time [3]. There is emerging evidence that changes in patterns of PA and SB are independently related to factors of cardiometabolic health including the metabolic syndrome [2, 4]. Meta-analysis data revealed that spending high amounts of time engaging in SB increased the odds of having the metabolic syndrome by 73% compared to spending low amounts of time engaging in SB [4].

The association between leisure-time SB and factors of cardiometabolic health depends on the type of SB and the age of individuals [5]. Many studies have focused on television [TV] time or time spent using a computer and their associations with cardiovascular health [2, 6]. Few studies have analyzed associations between other SBs such as reading or socializing in leisure time and cardiometabolic risk factors [7–12]. Time spent watching TV was found to be positively associated with obesity [10], type II diabetes [7], overweight [8, 9], or individual cardiometabolic biomarkers [10–12]. The evidence for using a computer in leisure time is inconsistent [7–12]. No associations have been found between other SBs such as reading or socializing in leisure time and cardiometabolic risk factors [7, 8].

A growing body of literature recommends using a cluster of continuous cardiometabolic risk factors instead of a binary definition of the metabolic syndrome [13, 14]. The reasons include (i) cardiovascular risk increases progressively with an increasing number of metabolic syndrome risk factors, and (ii) using a continuous risk score increases statistical power [14].

Current evidence has suggested that a greater increase in overall sedentary time is associated with a greater increase in clustered cardiometabolic risk in adults at high risk of developing type II diabetes [15] or in a population-based sample [16]. Nevertheless, to the best of our knowledge, no study has examined a broader range of screen-based and other leisure-time SBs and their associations with clustered cardiometabolic risk. One study has analyzed associations between SBs in leisure time and two clustered cardiometabolic risk scores over a 2-year follow-up period among adults at increased cardiometabolic risk [17]. A risk score of developing type II diabetes (Atherosclerosis Risk in Communities study) and a score of developing fatal cardiovascular disease (Systematic Coronary Risk Evaluation) was used. The findings suggested no associations between time spent watching TV, using a computer, or reading and individual cardiometabolic risk factors or clustered cardiometabolic risk scores [17].

Given the ubiquitous nature of prolonged SB in leisure time, a deeper understanding is needed about whether behaviors are associated with clustered cardiometabolic risk in order to develop adequate prevention strategies. The present study aimed to examine associations between leisure-time SBs (watching TV, using the computer, and playing computer games, reading, doing household tasks, caring for others, pursuing hobbies, and socializing) and clustered cardiometabolic risk in apparently healthy adults.

## Methods

### Selection of participants

Participants were recruited from a sample of 1165 individuals aged 40 to 75 years who had been recruited in general medical practices, job agencies, or via a health insurance company between June 2012 and December 2013 [18], and gave consent to be contacted again (95%). Of those, 513 persons fulfilled the following eligibility criteria: no history of cardiovascular event (myocardial infarction, stroke) or vascular intervention, age ≥ 40 and ≤ 65 years, self-reported body mass index ≤ 35 kg/m^2^, and residency in a pre-defined zip-code area. A total of 401 persons were contacted and offered participation in a study that aimed to assess the feasibility of a tailored counselling letter intervention to increase PA and to reduce sedentary time.

**Fig. 1:**
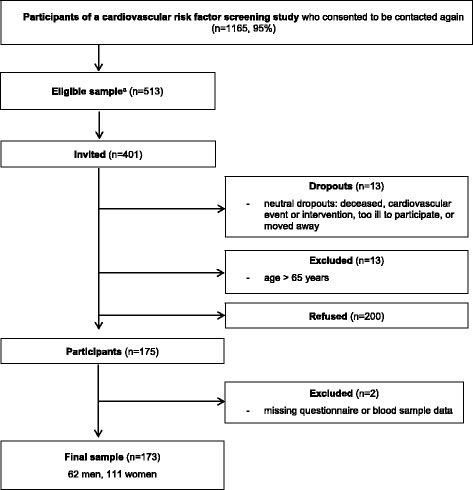
Flow of participation. ^a^ Eligibility criteria: no history of cardiovascular event (myocardial infarction, stroke), or vascular intervention, body mass index ≤ 35 kg/m^2^, residency in a pre-defined zip-code area, and age ≥ 40 and ≤ 65 years

Among those who had been offered study participation, 175 persons gave written informed consent and agreed to attend a cardiovascular examination program including the following: giving a blood sample, standardized measurement of blood pressure, waist circumference, body weight, and height at the study examination center, and completing a paper-pencil questionnaire on PA and leisure-time SB. Data were collected between February and July 2015. Two participants were excluded from analysis due to missing questionnaire or blood sample data. Our final sample comprised 173 adults (Fig. 1).

The study was approved by the clinical ethical committee of the University Medicine Greifswald (protocol number BB 002/15a).

### Measures

#### Clustered cardiometabolic risk score

According to the definition of the American Heart Association and the National Heart, Lung, and Blood Institute [19], six cardiometabolic risk factors were assessed: waist circumference [WC], systolic blood pressure [SBP], diastolic blood pressure [DBP], glucose, plasma triglycerides, and high-density lipoprotein cholesterol [HDL-C].

WC was measured midway between the lowest rib and the iliac crest using an inelastic tape. Blood pressure was measured after a five-minute resting period three times at the right arm in a seated position using a digital blood pressure monitor (705IT, Omron Corporation, Tokyo, Japan). The first and the second reading were followed by another resting period of 3 min each. For analyses, the means of the second and third measurements of SBP and DBP were used. If an antihypertensive medication within the last 12 months was reported, we added 10 mmHg to the original observed value of SBP and DBP [20]. Non-fasting blood samples were taken, and plasma triglycerides, HDL-C, and glucose were determined by standard methodology at the Institute for Clinical Chemistry and Laboratory Medicine of the University Medicine Greifswald. Values of plasma triglycerides were converted into corresponding fasting values by subtracting 3.7% of the triglyceride value per hour of non-fasting values. This calculation was made up to 8 h for men and up to 7 h for women [21].

The clustered cardiometabolic risk score [CMRS] was calculated according to Knaeps et al. [16] by the following method: glucose, triglycerides, and HDL-C were normalized (log_10_) due to their strongly skewed distributions. Each cardiometabolic variable was standardized by using sex-specific sample means and standard deviations [SD]. We standardized the scores of WC, SBP, DBP, glucose, triglycerides, and HDL-C. Because the standardized HDL-C is inversely related to metabolic risk, it was multiplied by − 1. CMRS was created by summing the standardized values of the six cardiometabolic risk factors and dividing the sum by 6 to account for the number of variables included. A higher CMRS indicates a higher cardiometabolic risk.

#### Sedentary behavior

We used a modified version of the “last 7-d sedentary behavior questionnaire” ([SIT-Q-7d], Section 5: Screen time and other activities) [22] to quantify the amount of time spent in different leisure-time SBs: watching TV, using a computer, playing computer games, reading, doing household tasks, caring for others, pursuing hobbies, and socializing. Because playing computer games was rarely present in our sample (*n* = 25), we summed time spent on using a computer and on playing computer games. Average time per day in hours (h/ day) was calculated.

#### Covariates

Sex, age, living in a partnership (yes/ no), and employment status (employed/ unemployed) were used as covariates. To account for other health-related confounders [2], leisure-time PA in Metabolic equivalent of task [MET]-hours per week and time spent traveling in a motor vehicle in minutes per day were assessed using the International Physical Activity Questionnaire [IPAQ] [23] and calculated according to the IPAQ protocol. The leisure-time domain includes walking, PA on a moderate-intensity level, and PA on a vigorous-intensity level. Especially prolonged time spent sitting in cars has shown to be associated with a more-adverse cardiometabolic risk profile [24]. Therefore, the single item of the IPAQ traveling in a motor vehicle was used as covariate.

### Statistics

Descriptive participant characteristics (mean [M] with SD and median [Med] with interquartile range [IQR]) were calculated. Leisure-time SB and PA variables were square root transformed to account for their right-skewed distributions. The constant 1 was added to the original value in order to anchor the variables at a place where square root transformation will have the optimal effect [25].

We performed multiple imputation using chained equations (m = 20 imputed datasets) to account for missing values. The six cardiometabolic risk factors included in the outcome variable (CMRS), the predictor variables (watching TV, using a computer, reading, and socializing), socio-demographic covariates (sex, age, living in a partnership, and employment status), PA covariates (leisure-time PA and traveling in motor vehicles), and auxiliary variables (e.g., body mass index, total cholesterol, glycated hemoglobin, low-density lipoprotein cholesterol) were considered for imputation models. To handle skewed continuous variables, we used the predictive mean matching method in the imputation procedure [26].

For analyzing associations of leisure-time SB with CMRS, we applied ordinary least-squares [OLS] regression. In regard of heteroscedasticity, we used robust standard errors estimations. Furthermore, we applied quantile regression [QR]. This method is not influenced by outliers in the distribution of the outcome variable [27] and served to explore the association of different leisure-time SBs across the entire distribution of CMRS [28]. We examined associations of SB variables at the 25th, 50th, and 75th percentiles of CMRS.

For the OLS and QR analyses, adjustments were made for socio-demographics and for time spent in the other leisure-time SBs (model 1). Subsequently, we adjusted for time spent physically active in leisure time and for time spent traveling in motor vehicles (model 2). Additionally, OLS and QR analyses using complete cases of the leisure-time SB variables were calculated as a sensitivity analysis. All variables of OLS regression analyses were tested for normality and residuals were tested for homoscedasticity, linearity and independence. To diagnose multicollinearity, the variance inflation factor was calculated. Values over 5 were considered as an indication of multicollinearity [29]. *P*-values below 0.05 were considered statistically significant. Statistical analyses were performed using STATA v.14.1 software (Stata Corporation, College Station, TX) [30].

## Results

### Characteristics of participants

In our sample, the mean age was 54.4 years (SD = 6.2), 64% were women, 81% were employed, and 72% lived in a partnership (Table 1). The mean CMRS was − 0.0 (SD = 0.6) with a minimum value of − 1.3 and a maximum value of 1.6.

**Table 1:** Descriptive characteristics of the study sample (*n* = 173)

Variables	Number	Missing data %	Values
Socio-demographic variables			
Age, years (M, SD)	173	–	54.4 ± 6.2
Gender (n, %)			
Female	173	-	111 (64.2)
Employed (n, %)	170	2	138 (81.2)
Partnership (n, %)	173	–	124 (71.7)
Cardiometabolic variables			
WC, cm (M, SD)	172	1	91.6 ± 12.5
SBP, mmHg (M, SD)	164	5	131.2 ± 16.1
DBP, mmHg (M, SD)	164	5	80.7 ± 11.0
Non-fasting glucose, mmol/l (Med, IQR)	163	6	5.3 (4.9–5.8)
HDL-C, mmol/l (M, SD)	167	3	1.4 ± 0.4
Plasma triglyceride, mmol/l (Med, IQR)	151	13	1.1 (0.7–1.7)
CMRS (M, SD)	170	2	−0.0 ± 0.6
Leisure-time sedentary behavior variables			
Watching TV, h/day (Med, IQR)	165	5	2.5 (1.8–3.5)
Using a computer, h/day (Med, IQR)	142	18	0.4 (0.1–1.0)
Reading, h/day (Med, IQR)	160	8	0.5 (0.4–1.0)
Household tasks, h/day (Med, IQR)	154	11	0 (0–0.1)
Caring for others, h/day (Med, IQR)	140	19	0 (0–0.1)
Hobbies, h/day (Med, IQR)	153	12	0.1 (0–0.4)
Socializing, h/day (Med, IQR)	151	13	0.4 (0–1.0)
Physical activity variables			
Leisure-time physical activity, MET-h/week (Med, IQR)	145	16	15.6 (3.3–33.1)
Traveling in motor vehicles, min/day (Med, IQR)	165	5	30 (10–60)

*M* mean, *SD* standard deviation, *Med* median, *IQR* interquartile range, *WC* waist circumference, *SBP* systolic blood pressure, *DBP* diastolic blood pressure, *HDL-C* high-density lipoprotein cholesterol, *CMRS* clustered cardiometabolic risk score, *TV* television, *MET* metabolic equivalent of task

Presented are means and standard deviations for normally distributed variables, medians and interquartile ranges for non-normally distributed variables, and absolute values and percentages for categorical variables

### Leisure-time sedentary behaviors

Doing household tasks was reported by 53, caring for others by 36, and pursuing hobbies by 87 study participants. Thus, they were excluded from the analysis and we analyzed associations of time spent watching TV, using a computer, reading, and socializing in leisure time (*n* = 161, *n* = 112, *n* = 156, and *n* = 113, respectively, with values over zero) with CMRS.

In median, participants spent 2.5 h/ day (IQR: 1.8–3.5) watching TV, 0.4 h/ day (IQR: 0.1–1.0) using a computer, 0.5 h/ day (IQR: 0.4–1.0) reading, and 0.4 h/ day (IQR: 0.0–1.0) socializing during leisure time (Table 1).

### Associations between leisure-time sedentary behaviors and clustered cardiometabolic risk score

In both models, OLS regression revealed that there was a positive association between watching TV and CMRS (model 1: *b* = 0.27 [*CI*: 0.03; 0.52]; model 2: *b* = 0.30 [*CI*: 0.05; 0.56]). As shown in Table 2, QR analysis revealed that watching TV was positively associated with the 25th (model 1: *b* = 0.35 [*CI*: 0.07; 0.63]; model 2: *b* = 0.34 [*CI*: 0.09; 0.59]), the 50th (model 1: *b* = 0.32 [*CI*: 0.02; 0.62], model 2: *b* = 0.37 [*CI*: 0.07; 0.66]), and the 75th percentiles (model 2: *b* = 0.32 [*CI*: 0.01; 0.63]) of CMRS. Furthermore, the 50th (model 1: *b* = − 0.43 [*CI*: -0.79; − 0.07]) and the 75th percentiles (model 1: *b* = − 0.71 [*CI*: -1.27; − 0.14]) of CMRS revealed a negative association with using a computer. These significant associations disappeared after additionally adjusting for time spent physically active in leisure time and for time spent traveling in motor vehicles (model 2, 50th percentile: *b* = − 0.28 [*CI*: -0.81; 0.24]; model 2, 75th percentile: *b* = − 0.55 [*CI*: -1.10; 0.01]). There were no statistically significant associations between reading or socializing and CMRS. OLS and QR analyses using complete cases of the leisure-time SB variables yielded similar results (see Additional file 1: Table S1).

**Table 2:** Results of linear and quantile regression of multiply imputed data (*n* = 173)

	OLS		QR25		QR50		QR75	
CMRS^a^	*b* [95% CI]	*p* ^b^	*b* [95% CI]	*p* ^b^	*b* [95% CI]	*p* ^b^	*b* [95% CI]	*p* ^b^
Watching TV								
Model 1^c^	0.27* [0.03; 0.52]	0.029	0.35* [0.07; 0.63]	0.015	0.32* [0.02; 0.62]	0.039	0.26 [−0.09; 0.62]	0.143
Model 2^d^	0.30* [0.05; 0.56]	0.021	0.34** [0.09; 0.59]	0.008	0.37* [0.07; 0.66]	0.015	0.32* [0.01; 0.63]	0.041
Using a computer								
Model 1^c^	− 0.35 [−0.70; 0.00]	0.051	−0.15 [−0.67; 0.18]	0.251	−0.43* [−0.79; − 0.07]	0.019	−0.71* [− 1.27; − 0.14]	0.015
Model 2^d^	− 0.26 [−0.65; 0.13]	0.188	−0.26 [−0.66; 0.13]	0.191	−0.28 [−0.81; 0.24]	0.277	−0.55 [−1.10; 0.01]	0.052
Reading								
Model 1^c^	− 0.07 [−0.57; 0.42]	0.766	− 0.18 [−0.71; 0.35]	0.502	−0.21 [−0.95; 0.53]	0.573	0.06 [−0.72; 0.83]	0.888
Model 2^d^	− 0.17 [−0.68; 0.34]	0.515	−0.36 [−0.84; 0.13]	0.145	−0.33 [−1.17; 0.50]	0.429	−0.04 [−0.77; 0.70]	0.922
Socializing								
Model 1^c^	− 0.08 [−0.38; 0.21]	0.578	0.07 [−0.33; 0.48]	0.718	−0.09 [−0.44; 0.26]	0.616	−0.26 [−0.72; 0.19]	0.255
Model 2^d^	− 0.06 [−0.37; 0.24]	0.687	0.08 [−0.23; 0.38]	0.619	−0.07 [−0.46; 0.31]	0.708	−0.20 [−0.64; 0.24]	0.374

*OLS* ordinary least squares regression, *QR* quantile regression, *b* unstandardized regression coefficient, *CI* confidence interval, *TV* television

^a^ Presented are multiply imputed data using chained equations (m = 20 imputed datasets) to account for missing values

^b^ Based on robust standard errors, **p < 0.01, *p < 0.05

^c^ Model 1: Adjusted for socio-demographic (sex, age, partnership, and employment) and other leisure-time sedentary behavior variables

^d^ Model 2: Adjusted for socio-demographic (sex, age, partnership, and employment), leisure-time physical activity, traveling in motor vehicles, and other leisure-time sedentary behavior variables

## Discussion

There were two main findings of our study. First, watching TV was positively associated with CMRS. In addition, depending on the quantiles of CMRS, QR analysis revealed a negative association between computer time and CMRS. However, this association disappeared after adjusting for PA in leisure time and time spent traveling in motor vehicles. Second, no associations were present between reading or socializing and CMRS.

Our results suggest that study participants who spend higher amounts of time watching TV are at higher cardiometabolic risk than individuals with low levels of TV time. This association remained significant after adjusting for leisure-time PA and time spent traveling in motor vehicles. Furthermore, QR analyses revealed an association between computer time and CMRS that otherwise is hidden if using the mean of CMRS in OLS regression analysis. Among study participants in the medium and in the highest cardiometabolic risk group, higher amounts of time using a computer were associated with a more favorable cardiometabolic profile. However, this association disappeared after adjusting for leisure-time PA and time spent traveling in motor vehicles.

Our findings on associations between TV time and CMRS are in line with current evidence on associations between TV time and individual cardiometabolic risk factors [12, 10, 2]. Additionally, watching TV has been shown to be associated with lower energy expenditure [31], an increased intake of food with high energy density and overall unhealthy dietary habits [32, 33] compared with other sedentary activities such as using a computer. A combination of these factors may explain our findings [12, 10]. According to the QR result, time spent sedentary while using a computer may be differentially associated with CMRS. Whereas a population-based study suggested no association between using a computer in leisure time and individual risk factors of cardiometabolic health [11], Heinonen et al. [10] reported a positive association between computer time and WC as well as body mass index among women but not among men in a middle-aged sample. In contrast to these studies, our results of QR revealed a negative association between using a computer and CMRS among individuals in the medium and in the highest cardiometabolic risk group. After adjusting for two PA variables, the statistically significant association between using a computer and CMRS disappeared whereas the negative direction of the association remained. Thus, different behaviors in which individuals spend their time in a sitting or reclining posture may not influence the magnitude and direction of the association with clustered cardiometabolic risk in the same manner.

Our second finding adds to the literature that there seems to be no association between reading and socializing and clustered cardiometabolic risk. This finding is in line with previous studies that examined associations between time spent sedentary while reading or socializing and individual cardiometabolic health factors [7–12, 17]. The less time spent reading or socializing in leisure time might be an explanation for the findings in our sample. In addition, accuracy to recall across contexts may vary [2] in the sense that it may be easier for people to remember how long they have watched TV than how much time they have spent reading or in company with others [34].

Furthermore, we found that leisure-time SBs differed in their frequency of occurrence. Time spent sitting while doing household tasks, caring for others, or pursuing hobbies were less prevalent in our sample. Evidence suggests that different types of SB often co-occur compared to activities with higher energy expenditure (over 1.5 metabolic equivalents) [22], e.g. using the computer or doing household tasks while watching TV. Thus, it is important to consider not only the frequency of occurrence but also that of co-occurrence of leisure-time SB and how different types of leisure-time SB are linked to one another. Future studies should examine those patterns of leisure-time SB in detail and might include separate analyses for weekdays and weekends, because leisure-time SB patterns have been shown to vary between weekends and weekdays [35].

Some limitations of our study have to be discussed. First, subjects were assessed within a study aiming to test the feasibility of a tailored letter intervention regarding PA and leisure-time SB. The proportion of people who declined participation (53%) was high and a selection bias is likely. Thus, our findings may not be generalizable to the population as a whole. Second, there may be confounding variables such as diet, drinking habits, different activity patterns with certain energy expenditure during leisure-time SBs, sleep duration, or cardiorespiratory fitness that were not considered in our study. Third, we collected blood samples in the non-fasted state. Because levels of glucose or HDL-C are influenced by external factors like caloric intake or muscle activity [36], this may have implications for the clustered cardiometabolic risk. Although using fasting blood samples is recommended, there is evidence that using non-fasting blood samples is appropriate for decision making in the context of primary preventions regarding cardiovascular or cardiometabolic diseases [36, 37]. Fourth, we assessed leisure-time SB by self-report. Self-report assessments are sensitive to recall bias and social desirability [2]. In comparison to accelerometer measures of SB, self-report appears to capture different aspects of behaviors [38] because it provides information on the context. Keeping in mind that leisure-time SB is complex and includes multiple domains, dimensions, and correlates, there are still many methodological challenges of measuring leisure-time SB [39]. Finally, the design of our study does not allow for causal inference. To address this issue, more longitudinal studies are needed to understand the directionality of potential associations between leisure-time SBs and cardiometabolic health.

## Conclusions

Watching TV was positively associated with a clustered cardiometabolic risk score, while results of time spent using a computer revealed inconsistent findings. No associations were present between reading or socializing and clustered cardiometabolic risk.

Our findings suggest that different leisure-time SBs and their differential associations with cardiometabolic risk should be considered. This approach would address the needs (i) for behavior specific assessments, (ii) to develop relevant public health recommendations and guidelines to maintain or enhance adults’ health, and (iii) to encourage environmental and policy initiatives and interventions [2].

## Additional file

Additional file 1: **Table S1.** Results of linear and quantile regression of complete cases. (DOCX 20 kb)

## Abbreviations

CI: Confidence interval
CMRS: Clustered cardiometabolic risk score
DBP: Diastolic blood pressure
HDL-C: High-density lipoprotein cholesterol
IPAQ: International physical activity questionnaire
IQR: Interquartile range
M: Mean
Med: Median
MET: Metabolic equivalent of task
OLS: Ordinary least-squares regression
PA: Physical activity
QR: Quantile regression
SB: Sedentary behavior
SBP: Systolic blood pressure
SD: Standard deviation
SIT-Q-7d: Last 7-d sedentary behavior questionnaire
TV: Television
WC: Waist circumference
