# Supplementary material for: A cross-sectional analysis of the associations between leisure-time sedentary behaviors and clustered cardiometabolic risk
Source: BMC Public Health. 2018 Mar 6;18:327. doi: 10.1186/s12889-018-5213-3 (PMC5839009; doi:10.1186/s12889-018-5213-3)
Supplement: Supplementary file 1 — Table S1. Results of linear and quantile regression of complete cases. (DOCX 20 kb) [file 12889_2018_5213_MOESM1_ESM.docx]

**Supplemental Data File**

**S table 1.** Results of linear and quantile regression of complete cases

|  | OLS |  |  | QR25 |  |  | QR50 |  |  | QR75 |  |  |
| --- | --- | --- | --- | --- | --- | --- | --- | --- | --- | --- | --- | --- |
| **CMRS**^a,c^ | *b* [95% CI] | *p*^b^ |  | *b* [95% CI] | *p*^b^ |  | *b* [95% CI] | *p*^b^ |  | *b* [95% CI] | *p*^b^ |  |
| **Watching TV** | | | | | | | | | | | | |
| Model 1^c^ | 0.34* | 0.025 |  | 0.64*** | <0.001 |  | 0.42** | 0.004 |  | 0.34 | 0.078 |  |
|  | [0.04; 0.65] |  |  | [0.39; 0.89] |  |  | [0.13; 0.70] |  |  | [-0.04; 0.72] |  |  |
| Model 2^d^ | 0.35 | 0.052 |  | 0.53* | 0.013 |  | 0.41* | 0.010 |  | 0.14 | 0.616 |  |
|  | [-0.00; 0.70] |  |  | [0.12; 0.95] |  |  | [0.10; 0.72] |  |  | [-0.41; 0.69] |  |  |
| **Using a computer** | | | | | | | | | | | | |
| Model 1^c^ | -0.30 | 0.109 |  | -0.13 | 0.492 |  | -0.47** | 0.008 |  | -0.57* | 0.016 |  |
|  | [-0.67; 0.07] |  |  | [-0.50; 0.24] |  |  | [-0.82; -0.13] |  |  | [-1.03; -0.11] |  |  |
| Model 2^d^ | -0.25 | 0.239 |  | -0.15 | 0.579 |  | -0.32 | 0.104 |  | -0.37 | 0.328 |  |
|  | [-0.66 0.17] |  |  | [-0.67; 0.38] |  |  | [-0.71; 0.07] |  |  | [-1.12; 0.38] |  |  |
| **Reading** | | | | | | | | | | | | |
| Model 1^c^ | -0.15 | 0.581 |  | -0.25 | 0.389 |  | -0.34 | 0.262 |  | -0.10 | 0.777 |  |
|  | [-0.67; 0.38] |  |  | [-0.82; 0.32] |  |  | [-0.93; 0.26] |  |  | [-0.77; 0.58] |  |  |
| Model 2^d^ | -0.16 | 0.599 |  | -0.29 | 0.420 |  | -0.33 | 0.375 |  | -0.07 | 0.874 |  |
|  | [-0.74; 0.43] |  |  | [-1.01; 0.43] |  |  | [-1.06; 0.40] |  |  | [-0.94; 0.80] |  |  |
| **Socializing** |  |  |  |  |  |  |  |  |  |  |  |  |
| Model 1^c^ | -0.10 | 0.497 |  | 0.03 | 0.863 |  | -0.12 | 0.404 |  | -0.06 | 0.766 |  |
|  | [-0.39; 0.19] |  |  | [-0.33; 0.39] |  |  | [-0.39; 0.16] |  |  | [-0.44; 0.32] |  |  |
| Model 2^d^ | -0.07 | 0.674 |  | -0.12 | 0.635 |  | 0.06 | 0.743 |  | -0.12 | 0.693 |  |
|  | [-0.41; 0.27] |  |  | [-0.62; 0.38] |  |  | [-0.28; 0.39] |  |  | [-0.75; 0.50] |  |  |

OLS, ordinary least squares regression; QR, quantile regression; b, unstandardized regression coefficient; CI, confidence interval; TV, television.

^a^ Presented are multiple imputed data using chained equations (m = 20 imputed datasets) to account for missing values.

^b^ Based on robust standard errors, *** p<0.001, ** p<0.01, * p<0.05.

^c^ Model 1 (n = 126): Adjusted for socio-demographic (sex, age, partnership, and employment) and other leisure-time sedentary behavior variables.

^d^ Model 2 (n = 108): Adjusted for socio-demographic (sex, age, partnership, and employment), leisure-time physical activity, traveling in motor vehicles, and other leisure-time sedentary behavior variables.
